# An Unlucky Four-Leaf Clover Stenosis With a Single Coronary Artery

**DOI:** 10.7759/cureus.51871

**Published:** 2024-01-08

**Authors:** Achraf Machraa, Lamyaa Bakamel, Jalal Tagueniti, Brahim Meftout, Pascal Goube

**Affiliations:** 1 Cardiac Catheterization Laboratory Unit, Department of Cardiology, Sud Francilien Hospital Center, Corbeil-Essonnes, FRA; 2 Clinical Cardiology Unit, Department of Cardiology, Sud Francilien Hospital Center, Corbeil-Essonnes, FRA

**Keywords:** cardiac computed tomography, congenital heart disease, single coronary artery, aortic stenosis, quadricuspid valve

## Abstract

The recognition of quadricuspid aortic valve has clinical significance as it leads to aortic valve dysfunction. Due to its frequent association with other congenital cardiac abnormalities, such as abnormally located coronary ostia, preoperative diagnosis is crucial. We present the case of a unique association of quadricuspid aortic valve stenosis with a single coronary artery.

## Introduction

Aortic valve (AV) anatomical variations, such as the unicuspid, bicuspid, and quadricuspid valves, have been reported in the literature. Among these, the quadricuspid aortic valve (QAV) is the rarest with an estimated frequency of <0.05% [1]. It is usually an isolated malformation, and adult patients generally present with gradual aortic regurgitation whereas aortic valve stenosis is uncommon [1,2]. It is often associated with other congenital malformations and up to 10% of patients with a QAV have been reported to have coronary anomalies [3,4].

The present report seeks to provide insights into a unique association and shed light on the importance of a comprehensive evaluation of the valve and coronary arteries and their relation with adjacent structures for preoperative planning.

## Case presentation

The patient was a 74-year-old woman. She was taking aspirin and fenofibrate for a transient ischemic attack and dyslipidemia and had quit smoking approximately four years earlier. She presented to her physician with worsening dyspnea and chest pain on exertion. Her dyspnea was classified as New York Heart Association functional class II-III, the chest pain was mainly a feeling of pressure and lasted for a few minutes. She was referred for cardiac evaluation because of a grade 4/6 aortic systolic murmur with no peripheral congestive signs. Her blood pressure and pulse rate were 115/59 mmHg and 70 beats/minute, respectively.

**Figure 1:**
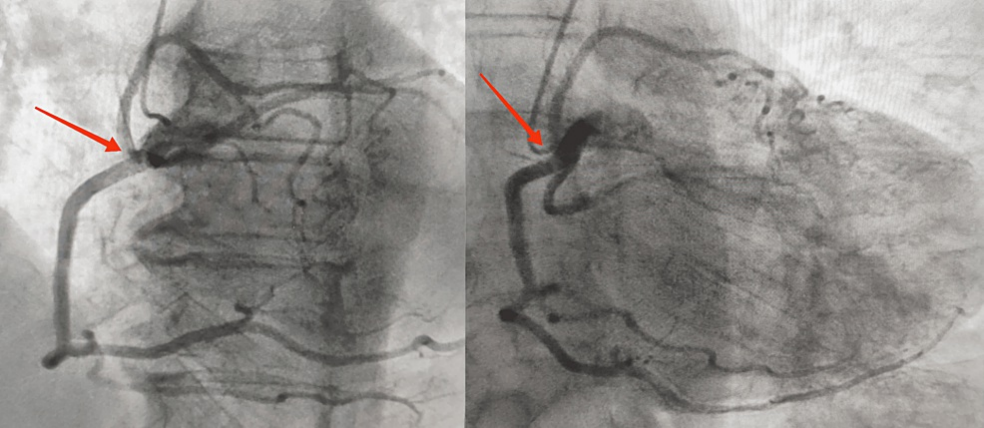
Coronary angiography showed a single coronary artery arising from the right sinus of Valsalva (Red arrow).

Two-dimensional transthoracic echocardiography (TTE) showed concentric left ventricular (LV) hypertrophy with a normal LV function, very severe calcified aortic stenosis (peak velocity of 5.4 m/s, mean gradient of 80 mmHg and a calculated aortic valve (AV) area of 0.5 cm²), no aortic regurgitation, and a normal-size ascending aorta. AV leaflet morphology was not well visualized on TTE. No other significant abnormalities were noted. The coronary angiography showed a single coronary artery arising from the right sinus of Valsalva. From the proximal part of this anomalous trunk, the left anterior descending artery (LAD) and the circumflex (Cx) arose (Figure 1). There were no atherosclerotic lesions in the coronary arteries.

**Figure 2:**
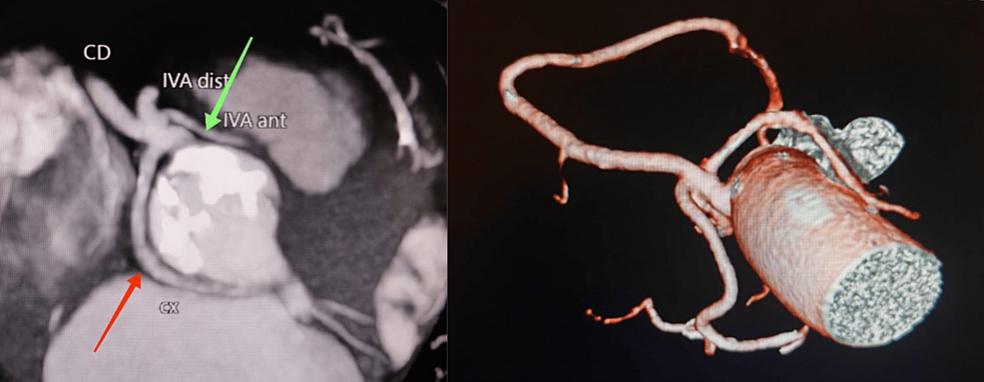
Multidetector computed tomography showing all three major coronaries arising from the right sinus via common ostium. The proximal LAD has an interarterial course (Green arrow). The Cx has a retroaortic course (Red arrow). LAD: left anterior descending artery; Cx: circumflex

**Figure 3:**
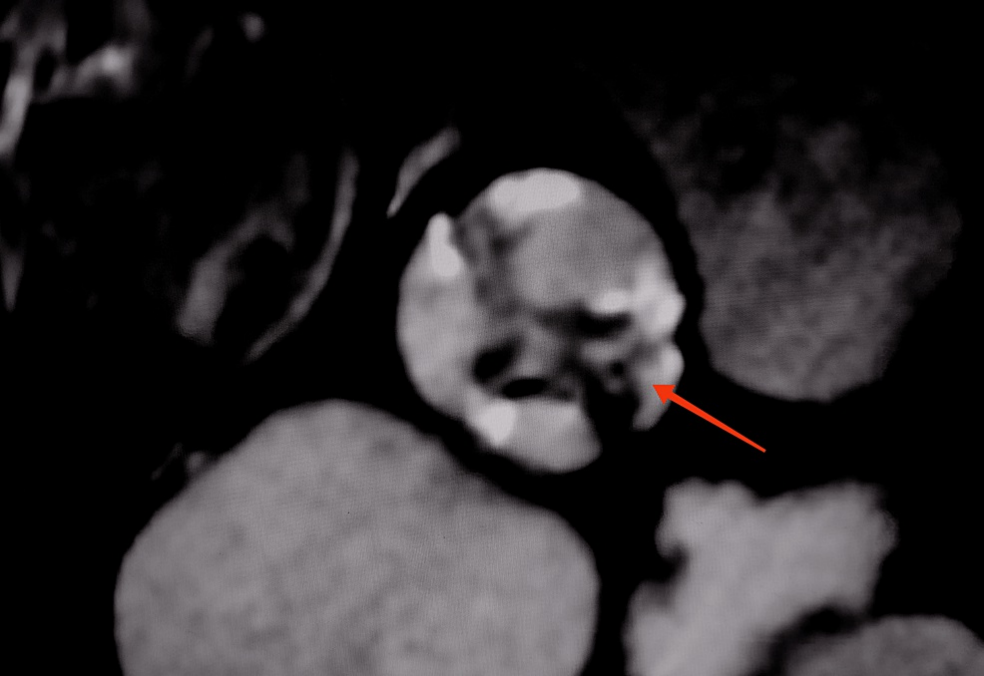
Multidetector computed tomography revealed a quadricuspid aortic valve with two equal-sized large cusps and two unequal smaller cusps (Red arrow).

In order to better define the course of the LAD and the Cx, we performed a multidetector computed tomography (MCT) (Figure 2) which confirmed that from the proximal part of this single coronary artery arises a very thin branch (proximal LAD) with an interarterial course before reaching the proximal part of the anterior interventricular sulcus. The second branch (distal LAD), which is large, passes in front of the pulmonary artery to reach the distal part of the anterior interventricular sulcus. The third branch (Cx) has a retroaortic course before reaching the left coronary sulcus. The right coronary artery (RCA) has a normal course, it passes along the right coronary sulcus. The MCT also revealed a QAV with two equal-sized large cusps and two unequal smaller cusps (Figure 3).

Taking into account the severity of the aortic stenosis and a low mortality risk based on a Society of Thoracic Surgery score of 1.19%, the heart team recommended performing a surgical isolated AV replacement with a biological prosthesis with no intervention on the coronary artery. No complications occurred post implantation and the postoperative TTE displayed a normally functioning prosthesis without paravalvular leakage.

At the six-month follow-up, the patient remains asymptomatic. Transesophageal echocardiogram (TEE) demonstrated a normally functioning bioprosthesis. No intervening adverse events occurred.

## Discussion

QAV is a rare congenital heart valve disease with an estimated incidence of 0.006-0.043% based on autopsy and echocardiography data [1]. QAV commonly occurs as an isolated congenital anomaly, although concomitant cardiac lesions coexist in 18% of the cases [3,5]. Coronary artery anomalies are reported in 10% of QAV cases [3]. Reported coronary artery anomalies include abnormalities in the origin, course of one of the coronary arteries, coronary-pulmonary artery fistula, and giant coronary artery aneurysm [6,7].

Amongst the cases with functional impairment of the aortic valve, aortic regurgitation is the most common affecting more than 75% of cases. Less than 1% of patients have isolated aortic stenosis, while 8-25% of patients have combined regurgitation and stenosis [8]. The case described in this report, of a woman with isolated severe aortic stenosis with a quadricuspid valve and a single coronary artery, is, therefore, a unique presentation.

**Figure 4:**
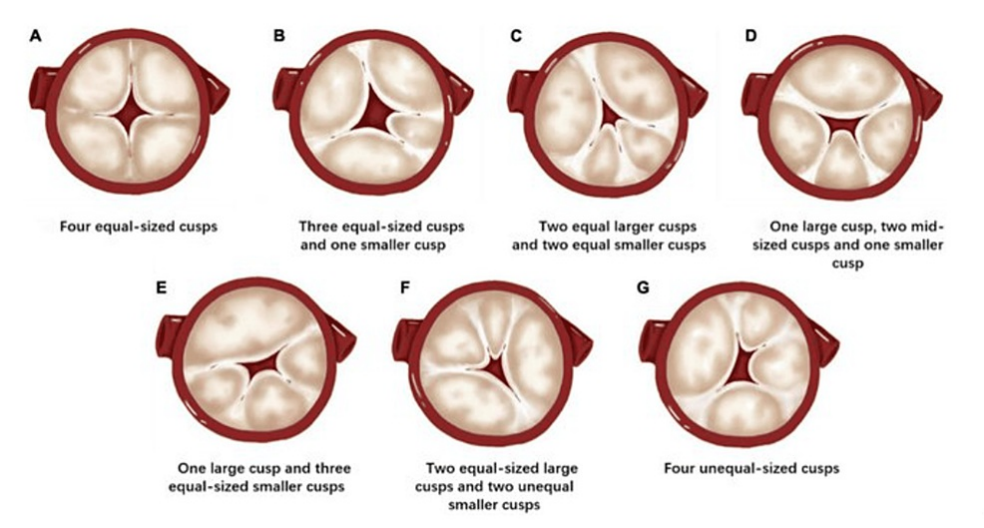
Hurwitz and Roberts' classification of the quadricuspid aortic valve Figure source: Liu et al., 2022 [11]; open-access distributed under the terms of the Creative Commons Attribution License CC BY 4.0 Deed, Attribution 4.0 International

As described by Hurwitz and Roberts, QAVs were classified into seven subtypes based on leaflet size and distribution (Figure 4) [9]. Approximately 73% of all QAV cases are Types A and B. According to Nakamura et al., there are other classification systems that are based on the location of the accessory cusps [10]. The current patient was classified as having Hurwitz type F and Nakamura type I.

Specific recommendations for the management of patients with QAVs and coronary artery anomalies have not been published. Hemodynamic complications such as severe aortic stenosis or regurgitation may require valve replacement or repair. Patients with a QAV that primarily causes aortic regurgitation may occasionally undergo valve repair (tricuspidization) [4], but surgical or transcatheter valve replacement is typically the preferred treatment for patients with aortic stenosis related to QAV [5]. There are very few documented cases of stenosed QAV that were successfully treated with a transcatheter approach [12].

The single coronary artery originating from the right sinus of Valsalva is a rare but well-known anatomical variant, which can occur in approximately 0.024-0.04% of the population [13]. A coronary anomaly is generally considered to be at high ischemic risk if it courses between the aorta and the pulmonary artery and ectopically originates from the opposite sinus. In the current patient, the Cx had a retroaortic course. The distal LAD passed in front of the pulmonary artery, while the proximal LAD was very thin and had an interarterial course. It means that only the course of the proximal LAD was at high risk, and it was not associated with other high-risk anatomic features. However, although this finding was classified as malignant, the heart team decided to be conservative and not intervene on the coronary arteries considering the small size of the vessel with an interatrial course and the small area supplied by the same vessel.

## Conclusions

The majority of QAV cases are still been diagnosed intraoperatively. Current diagnostic technology has given us comprehensive knowledge about cardiac structural abnormalities, particularly during the preoperative work-up phase. The current case illustrates the importance of a comprehensive evaluation of the valve and coronary arteries and the central role of the Heart Team concept in decision-making.

## References

[1] Tsang MY, Abudiab MM, Ammash NM, Naqvi TZ, Edwards WD, Nkomo VT, Pellikka PA (2016). Quadricuspid aortic valve: characteristics, associated structural cardiovascular abnormalities, and clinical outcomes. Circulation.

[2] Jagannath AD, Johri AM, Liberthson R, Larobina M, Passeri J, Tighe D, Agnihotri AK (2011). Quadricuspid aortic valve: a report of 12 cases and a review of the literature. Echocardiography.

[3] Tutarel O (2004). The quadricuspid aortic valve: a comprehensive review. J Heart Valve Dis.

[4] Idrees JJ, Roselli EE, Arafat A, Johnston DR, Svensson LG, Sabik JF 3rd, Pettersson GB (2015). Outcomes after repair or replacement of dysfunctional quadricuspid aortic valve. J Thorac Cardiovasc Surg.

[5] Yuan SM (2016). Quadricuspid aortic valve: a comprehensive review. Braz J Cardiovasc Surg.

[6] Agarwal A, Port S, Allaqaband S, Tajik AJ (2013). A unique case of quadricuspid aortic valve with coronary artery and descending aorta-to-pulmonary artery fistulae. Circulation.

[7] Okamoto M, Tomomori S, Kinoshita H (2013). An extremely large coronary aneurysm associated with a quadricuspid aortic valve in an adult patient. Intern Med.

[8] Lin Y, Yin K, Wang Y (2018). Clinical characteristics and surgical outcomes of dysfunctional quadricuspid aortic valve. J Surg Res.

[9] Hurwitz LE, Roberts WC (1973). Quadricuspid semilunar valve. Am J Cardiol.

[10] Nakamura Y, Taniguchi I, Saiki M, Morimoto K, Yamaga T (2001). Quadricuspid aortic valve associated with aortic stenosis and regurgitation. Jpn J Thorac Cardiovasc Surg.

[11] Liu Y, Zhai M, Mao Y (2022). Transcatheter aortic valve replacement in patients with quadricuspid aortic valve in a single center. Front Cardiovasc Med.

[12] Ibrahim M, Wattanakit K, Barzallo M, Mungee S (2018). Quadricuspid aortic valve stenosis: expanding our experience in transcatheter aortic valve implantation. J Invasive Cardiol.

[13] Desmet W, Vanhaecke J, Vrolix M, Van de Werf F, Piessens J, Willems J, de Geest H (1992). Isolated single coronary artery: a review of 50,000 consecutive coronary angiographies. Eur Heart J.

